# The Relationship between Prohibitin 1 Expression, Hepatotoxicity Induced by Acetaminophen, and Hepatoprotection by S-Adenosylmethionine in AML12 Cells

**DOI:** 10.4014/jmb.2207.07035

**Published:** 2022-09-26

**Authors:** Eunhye Cho, Soohan Jung, Jina Kim, Kwang Suk Ko

**Affiliations:** 1 Department of Nutritional Science and Food Management, Ewha Womans University, Seoul 03760, Republic of Korea; 2 Department of Integrated Biomedical and Life Science, Korea University, Seoul 02841, Republic of Korea; 3 Department of Human Biology, University of Southern California, Los Angeles, CA 90089, USA; 4 Graduate Program in System Health Science and Engineering, Ewha Womans University, Seoul 03760, Republic of Korea

**Keywords:** Prohibitin 1, acetaminophen, S-adenosylmethionine, hepatotoxicity

## Abstract

Prohibitin 1 (Phb1) is a pleiotropic protein, located mainly in the mitochondrial inner membrane and involved in the regulation of cell proliferation and the stabilization of mitochondrial protein. Acetaminophen (APAP) is one of the most commonly used over-the-counter analgesics worldwide. However, at high dose, the accumulation of N-acetyl-p-benzoquinone imine (NAPQI) can lead to APAP-induced hepatotoxicity. In this study, we sought to understand the regulation of mRNA expression in relation to APAP and GSH metabolism by Phb1 in normal mouse AML12 hepatocytes. We used two different Phb1 silencing levels: high-efficiency (HE, >90%) and low-efficiency (LE, 50-60%). In addition, the siRNA-transfected cells were further pretreated with 0.5 mM of S-adenosylmethionine (SAMe) for 24 h before treatment with APAP at different doses (1-2 mM) for 24 h. The expression of APAP metabolism-related and antioxidant genes such as Cyp2e1 and Ugt1a1 were increased during SAMe pretreatment. Moreover, SAMe increased intracellular GSH concentration and it was maintained after APAP treatment. To sum up, Phb1 silencing and APAP treatment impaired the metabolism of APAP in hepatocytes, and SAMe exerted a protective effect against hepatotoxicity by upregulating antioxidant genes.

## Introduction

Acetaminophen (APAP) is a widely used over-the-counter drug that acts as an antipyretic and analgesic. However, it is also a major cause of drug-induced liver injury. According to the American Association of Poison Control Centers (AAPCC), more than 120,000 people were exposed to toxic concentrations of APAP, and 342 of them were associated with death from APAP overdose [1]. At therapeutic doses, approximately 50‒70% of APAP is conjugated with glucuronide, and 25‒35% is sulfated before being excreted through urine. The remaining 5‒10% is converted into the toxic metabolite, N-acetyl-p-benzoquinone imine (NAPQI), by cytochrome P450 family 2 subfamily E member 1 (CYP2E1) [2, 3]. A small amount of NAPQI is eliminated quickly by reacting with hepatic glutathione (GSH); however, during an APAP overdose, the glucuronide and sulfate pathways are saturated, resulting in GSH depletion in hepatocytes and consequent accumulation of NAPQI in the liver [4, 5]. NAPQI can form protein adducts by covalently binding to hepatocellular proteins inside the mitochondria to induce hepatotoxicity [6].

Prohibitin 1 (PHB1) is a pleiotropic protein in mammalian cells involved in the regulation of cell proliferation, differentiation, and apoptosis [7]. It is mainly present in the mitochondrial inner membrane and is also ubiquitously expressed in the plasma membrane and the nucleus [8]. PHB1 stabilizes mitochondrial protein and is essential for normal mitochondrial development [9]. In normal hepatocytes, PHB1 helps cells to grow and function normally. Studies have shown that in a liver-specific Phb1 knockout (Phb1 KO) mouse, H19 and Igf2 genes were upregulated and controlled by the imprinting control region containing CCCTC-binding transcription factor binding sites [7]. H19 and Igf2 levels increased by 8-fold and 20-fold, respectively, compared to those of the control group. In addition, Phb1 KO mice showed reduced GSH production, which leads to persistent oxidative stress [10]. Therefore, the decrease in PHB1 protein may be closely related to the liver damage induced by APAP through diminished antioxidant mechanism.

S-Adenosylmethionine (SAMe) is an important molecule involved in multiple metabolic cellular reactions, such as the synthesis of GSH, as a precursor and principal methyl donor [11]. SAMe is synthesized from methionine and is involved in three key metabolic pathways: transmethylation, transsulfuration, and polyamine synthesis. Up to 85% of all methylation reactions and regulation of SAMe levels for metabolic homeostasis occur in the liver [12]. Additionally, SAMe can regulate hepatocyte growth, death, and differentiation [13]. As SAMe is a precursor for the synthesis of cysteine and GSH, it can increase intracellular GSH concentration by its conversion to GSH in hepatic mitochondria, thus normalizing the fluidity of the inner mitochondrial membrane [14].

Although hepatotoxicity induced by APAP and hepatoprotective effects of SAMe have been studied, no research has been conducted on the relevance of Phb1 expression levels for hepatotoxicity caused by APAP. Therefore, we examined the relationship between Phb1 expression and APAP-induced hepatotoxicity as well as the protective effects of SAMe against this hepatotoxicity.

## Materials and Methods

### Cell Culture

AML12, a normal murine hepatocyte cell line from the American Type Culture Collection (USA), was cultured in a 1:1 mixture of Dulbecco’s modified Eagle’s medium and Ham’s F12 medium (GE Healthcare, USA) supplemented with 10% fetal bovine serum (Corning, Inc., USA), 1% penicillin–streptomycin (Gibco, USA), 0.5 mM sodium pyruvate (Sigma-Aldrich, USA), 0.6 g of sodium bicarbonate (Daejung Chemicals Co., Korea), 40 ng/ml dexamethasone (Sigma-Aldrich), and a mixture of 5 μg/ml insulin, 5 μg/ml transferrin, and 5 ng/ml selenium (ITS, Sigma-Aldrich). The culture was then incubated at 37°C in a 5% CO_2_ humidified incubator.

### Small Interfering RNA (siRNA) Transfection

AML12 cells were seeded and transfected in 6-well plates 0.2 × 10^6^ cells/well) with 12 nM or 36 nM Phb1 siRNA (Bioneer, Korea), supplemented with Opti-MEM medium (Gibco) and Lipofectamine RNAiMAX (Invitrogen, USA) at 37°C in a 5% CO_2_ humidified incubator for 24 h. For different Phb1 silencing levels, two types of siRNA were used. One is a high-efficiency (HE) siRNA that lowered the Phb1 expression level to 10%, and the other is a low-efficiency (LE) siRNA that lowered the Phb1 expression level to 50‒60% compared to that of normal hepatocytes.

### S-Adenosylmethionine (SAMe) and Acetaminophen (APAP) Treatment

SAMe (Samoh Pharm Co., Ltd., Korea) was dissolved in 0.2 M tris(hydroxymethyl)aminomethane (Tris). APAP (Sigma-Aldrich) was dissolved in 99.9% ethanol to obtain concentrations of 1‒2 mM. Cells were pretreated with 0.5 mM SAMe for 24 h before treatment with APAP at different doses for 24 h at 37°C in a 5% CO_2_ humidified incubator.

### Cell Viability Assay

The number of viable cells was determined using the Quanti-Max WST-8 Cell Viability Assay Kit (Biomax, Korea) according to the manufacturer’s instructions.

### RNA Isolation and Quantitative Polymerase Chain Reaction (qPCR)

Total RNA was isolated using TRIzol reagent (Lifr technologies, Inc., USA) and reverse-transcribed to complementary DNA (cDNA) by using the RevertAid First Strand cDNA Synthesis Kit (Thermo Fisher Scientific, USA), according to the manufacturer’s instructions. qPCR was performed by following the instructions accompanying the Maxima SYBR Green/ROX qPCR Master Mix (Thermo Fisher Scientific). The relative expression levels of the target mRNAs were normalized to the expression of β-actin, an internal control, by using the 2–ΔΔCt method. All data were expressed relative to control values.

### Intracellular GSH Concentration

The concentration of GSH was estimated by reducing the total oxidized GSH using GSH reductase (Sigma-Aldrich).

### Statistical Analysis

All data are presented as mean ± standard error of the mean (SEM). Statistical analysis involved a one-way analysis of variance (ANOVA), followed by Duncan’s test using SAS 9.4 (SAS, Inc., USA). Differences were considered to be statistically significant at *p* < 0.05.

## Results

### Prohibitin 1 was silenced by siRNA transfection in AML12 cells

**Fig. 1:**
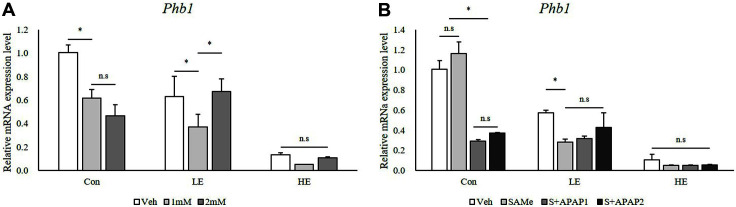
Effects of SAMe and APAP on relative mRNA levels of Phb1 in Phb1 siRNA-transfected AML12 cells. (**A**) APAP treatment; (**B**) SAMe + APAP treatment. An asterisk means significant difference between groups (*p* < 0.05). Each bar represents the mean ± standard error. Con, Control; LE, Low efficiency; HE, High efficiency; Veh, Vehicle; 1 mM, APAP 1 mM; 2 mM, APAP 2mM; SAMe, SAM 0.5 mM; S+APAP1, SAMe 0.5 mM+APAP 1 mM; S+APAP2, SAMe 0.5 mM+ APAP 2 mM.

To identify changes in Phb1 silencing after SAMe and APAP treatment, Phb1 mRNA levels were quantified by qPCR. Compared to the vehicle (Veh) in the control group, Phb1 expression in Veh in the LE group decreased to < 60%, whereas Phb1 expression in Veh in the HE group decreased to < 10% after Phb1 siRNA transfection (Figs. 1A and 1B).

### Cell viability was influenced by Phb1 silencing as well as treatment with APAP and SAMe

**Fig. 2:**
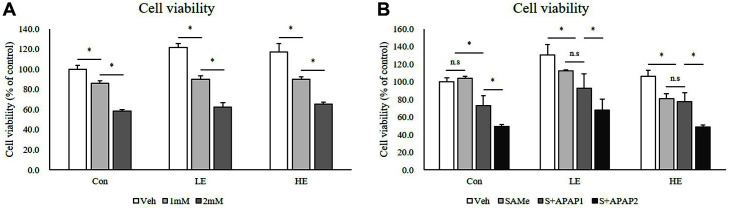
Effects of SAMe and APAP on cell viability in Phb1 siRNA-transfected AML12 cells. (**A**) APAP treatment; (**B**) SAMe + APAP treatment. An asterisk means significant difference between groups (*p* < 0.05). Each bar represents the mean ± standard error. Con, Control; LE, Low efficiency; HE, High efficiency; Veh, Vehicle; 1 mM, APAP 1 mM; 2 mM, APAP 2 mM; SAMe, SAM 0.5 mM; S+APAP1, SAMe 0.5 mM+APAP 1 mM; S+APAP2, SAMe 0.5 mM+ APAP 2 mM.

The effects of Phb1 silencing and of treatment with APAP and SAMe on cell viability showed that viability decreased as APAP concentration increased regardless of the Phb1 silencing level (Figs. 2A and 2B). For Veh in the LE group, cell viability was slightly higher than that observed for Veh in the control group. Consistent with the expected role of Phb1 as a tumor suppressor gene and regulator of cell proliferation, cell viability increased when Phb1 expression was 50% of the normal level.

### APAP Metabolism

**Fig. 3:**
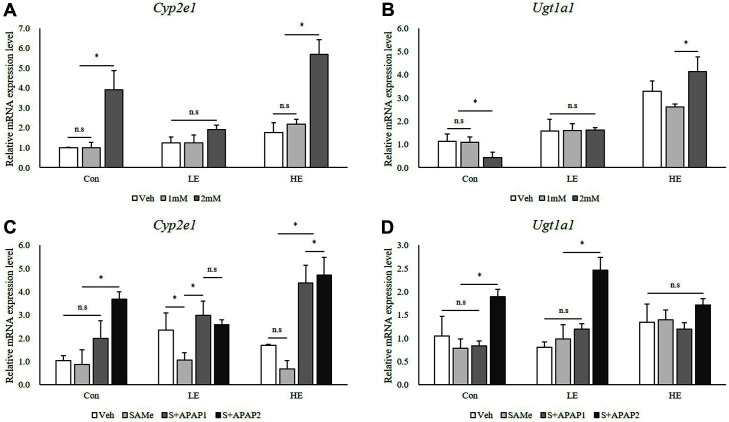
Effects of SAMe and APAP on relative mRNA levels of Cyp2e1 and Ugt1a1 in AML12 cells. (**A**) and (**B**) APAP treatment; (**C**) and (**D**) SAMe + APAP treatment. An asterisk means significant difference between groups (*p* < 0.05). Each bar represents the mean ± standard error. Con, Control; LE, Low efficiency; HE, High efficiency; Veh, Vehicle; 1 mM, APAP 1 mM; 2 mM, APAP 2 mM; SAMe, SAM 0.5 mM; S+APAP1, SAMe 0.5 mM+APAP 1 mM; S+APAP2, SAMe 0.5 mM+ APAP 2 mM.

Next, we measured the mRNA levels of APAP metabolism-related genes. An increase in Cyp2e1 expression was observed only within the control and Phb1 HE groups treated with 2 mM of APAP (Fig. 3A). Pretreatment with SAMe lowered the Cyp2e1 expression level that was upregulated in the APAP treatment groups (Fig. 3C). Ugt1a1 mRNA levels increased with APAP treatment in the Phb1 HE group (Fig. 3C) and further increased significantly in all groups upon treatment with 2 mM APAP and pretreatment with SAMe (Fig. 3D).

### Oxidative Stress

**Fig. 4:**
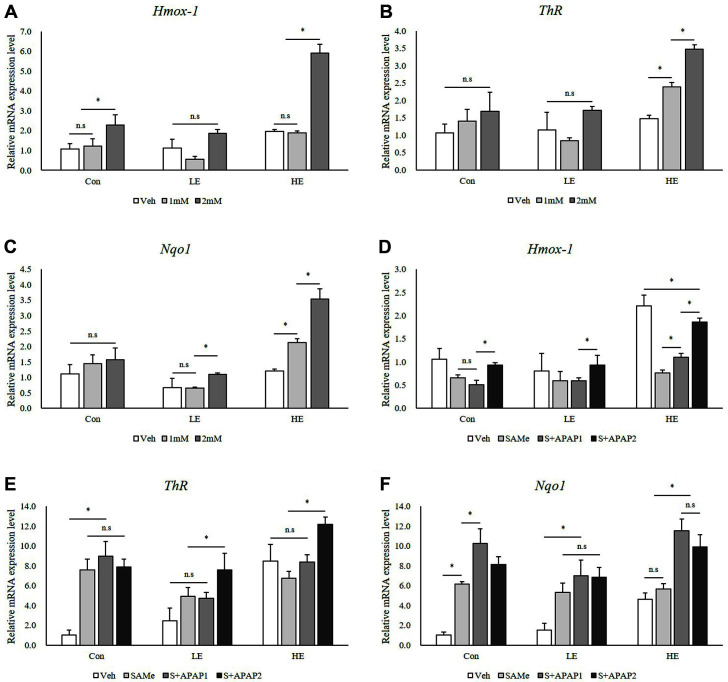
Effects of SAMe and APAP on oxidative stress-related genes in AML12 cells. (**A**), (**B**), and (**C**) APAP treatment; (**D**), (**E**), and (**F**) SAMe + APAP treatment. An asterisk means significant difference between groups (*p* < 0.05). Each bar represents the mean ± standard error. Con, Control; LE, Low efficiency; HE, High efficiency; Veh, Vehicle; 1 mM, APAP 1 mM; 2 mM, APAP 2 mM; SAMe, SAM 0.5 mM; S+APAP1, SAMe 0.5 mM+APAP 1 mM; S+APAP2, SAMe 0.5 mM+ APAP 2 mM.

To examine the protective mechanism against oxidative stress induced by APAP, we measured antioxidant gene expression and found that Hmox-1, ThR, and Nqo1 expression was upregulated in a dose-dependent manner by 2 mM of APAP in the Phb1 HE group (Figs. 4A-4C). The Hmox-1 expression was decreased by SAMe treatment in Phb1 HE group compared to Veh group (*p* < 0.05) (Fig. 4D). The mRNA levels of ThR and Nqo1 were significantly increased in the Phb1 HE groups after SAMe and APAP treatment (Figs. 4E and 4F).

### Intracellular GSH Concentration

**Fig. 5:**
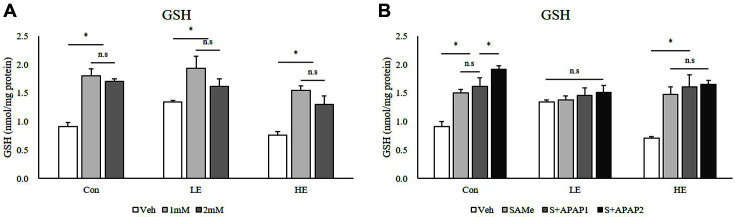
Effects of SAMe and APAP on intracellular GSH concentration in AML12 cells. (**A**) APAP treatment; (**B**) SAMe + APAP treatment. An asterisk means significant difference between groups (*p* < 0.05). Each bar represents the mean ± standard error. Con, Control; LE, Low efficiency; HE, High efficiency: Veh, Vehicle; 1 mM, APAP 1 mM; 2 mM, APAP 2 mM; SAMe, SAM 0.5 mM; S+APAP1, SAMe 0.5 mM+APAP 1 mM; S+APAP2, SAMe 0.5 mM+ APAP 2 mM.

To investigate the effects of Phb1 silencing and of treatment with SAMe and APAP on GSH concentration, intracellular GSH levels were measured. When treating Phb1 knockdown (KD) groups with APAP, GSH concentration decreased as APAP concentration increased (Fig. 5A). However, with SAMe pretreatment, the GSH-lowering effect of Phb1 silencing was not observed, and GSH concentration was maintained at levels similar to that observed without SAMe pretreatment (Fig. 5B).

### GSH Synthesis

**Fig. 6:**
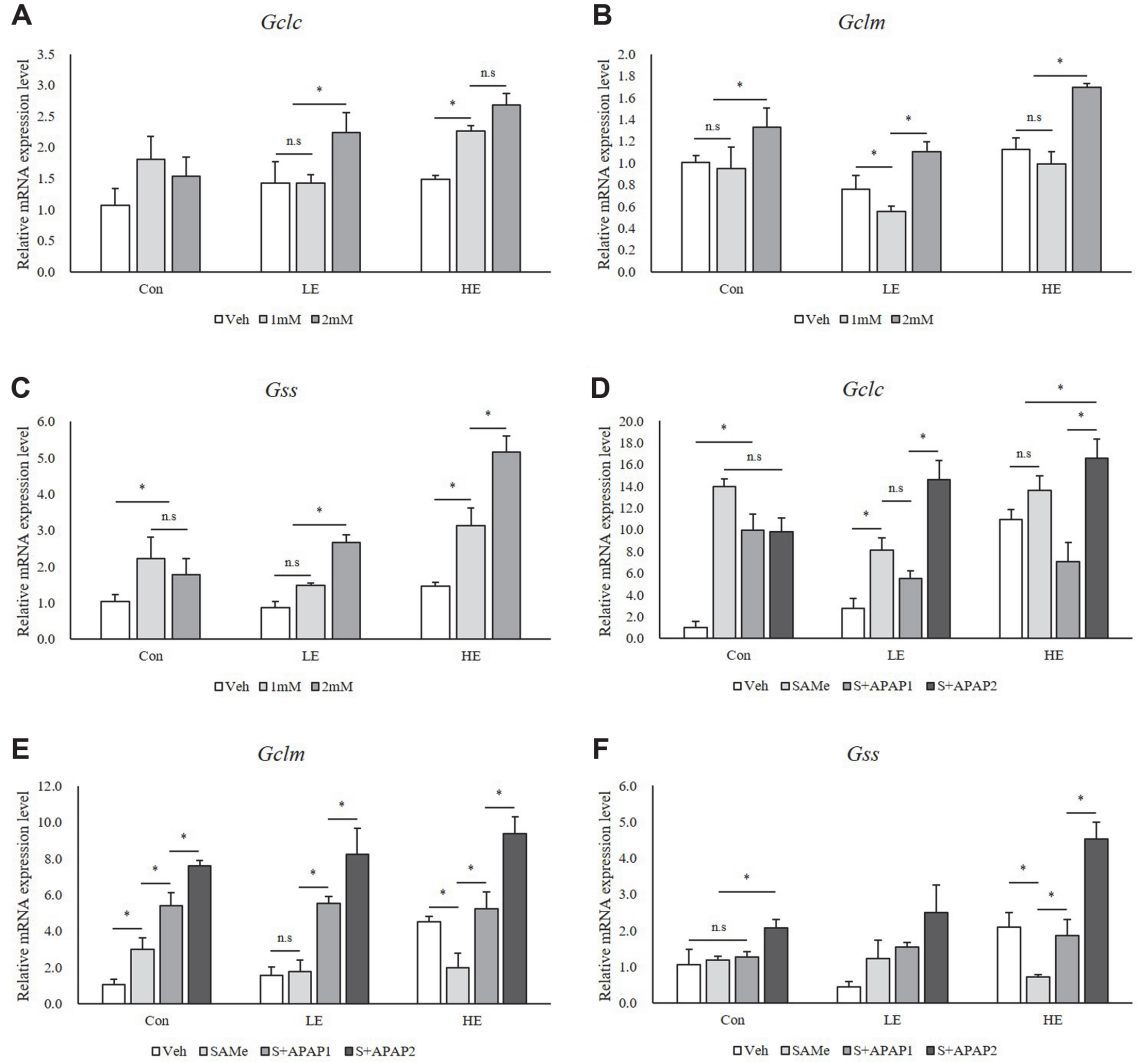
Effects of SAMe and APAP on GSH synthesis genes in Phb1 siRNA-transfected AML12 cells. (**A**), (**B**), and (**C**) APAP treatment; (**D**), (**E**), and (**F**) SAMe + APAP treatment. An asterisk means significant difference between groups (*p* < 0.05). Each bar represents the mean ± standard error. Con, Control; LE, Low efficiency; HE, High efficiency; Veh, Vehicle; 1 mM, APAP 1 mM; 2 mM, APAP 2 mM; SAMe, SAM 0.5 mM; S+APAP1, SAMe 0.5 mM+APAP 1 mM; S+APAP2, SAMe 0.5 mM+ APAP 2 mM.

The mRNA levels of Gclc and Gclm in the Phb1 HE group treated with 2 mM APAP were higher than those of the control group (Figs. 6A and 6B). Gss was upregulated in the Phb1 HE group when treated with 2 mM APAP (Fig. 6C). Similarly, although a general increase in Gclc mRNA levels was observed after treatment with both SAMe and APAP, the Phb1 KD group treated with 2 mM APAP showed a significant increase (Fig. 6D). Gclm was upregulated in all groups treated with 2 mM APAP (Fig. 6E), and Gss was significantly upregulated in the Phb1 HE group treated with 2 mM APAP (Fig. 6F).

## Discussion

As PHB1 is known to stabilize mitochondrial proteins, silencing of Phb1 may lead to mitochondrial dysfunction and oxidative stress [10]. In a previous study, liver-specific deletion of Phb1 caused liver injury including necrosis, apoptosis, swollen mitochondria, and fibrosis. In addition, liver-specific Phb1 KO mice exhibited upregulation of fibrogenesis-related genes and downregulation of xenobiotic metabolism-related genes, such as cytochrome P450, compared to wild-type mice [10]. Therefore, we investigated the effects of Phb1 expression on liver damage caused by a xenobiotic such as APAP to determine whether Phb1 deficiency might be an important factor in the prediction and treatment of liver injury.

In this study, Phb1 in AML12 cells was silenced with siRNA transfection to reduce its expression and the expression level of Phb1 was compared after APAP treatment. Compared to Veh in the control group, APAP treatment caused a decrease in Phb1 expression levels in the Con and LE groups (Figs. 1A and 1B). In contrast, the Phb1 HE group showed a decrease in Phb1 mRNA levels to ~10% of the normal levels, regardless of APAP treatment. Even after SAMe pretreatment, there were no significant changes in the expression levels of the groups (Fig. 1B). These results can be interpreted in two ways. First, even if Phb1 was only expressed at 50% of normal levels, it was not significantly affected by the reduction in its expression caused by APAP. Second, pretreatment with SAMe led to an increase in Phb1 expression in the control group without APAP, but led to no significant differences between the vehicle, SAMe-pretreated, and SAMe + APAP-treated hepatocytes in the Phb1 KD groups (LE and HE).

Cytochrome P450 (CYP) enzymes are responsible for the metabolism, in which CYP2E1 has been shown to play an important role, of most of the foreign chemicals [15, 16]. In this study, the mRNA levels of Cyp2e1 were significantly increased in the Phb1 HE group at 2 mM of APAP (Fig. 3A). However, the pretreatment with SAMe particularly affected the groups treated with APAP as Cyp2e1 expression increased after treatment with both SAMe and APAP, but was lower after treatment with only APAP. These results may indicate that SAMe exerted a protective effect by causing an increase in Cyp2e1 expression in the Phb1 HE group treated with 2 mM of APAP, as seen in Figs. 3A and 3C. This is most likely due to the higher viability that facilitated the detoxification of such a high concentration of APAP.

Most of the APAP administered in therapeutic doses is metabolized in the liver by UDP-glucuronosyltransferase (UGT) enzymes [17]. In this study, we showed that Ugt1a1 expression was increased after treatment with 2 mM of APAP in the Phb1 HE group (Fig. 3B). However, after SAMe pretreatment, a remarkable increase in Ugt1a1 expression was observed in the LE group (50‒60% Phb1 expression) treated with 2 mM of APAP (Fig. 3D), which may suggest that Phb1 expression should be maintained to at least 50% of the control for the protective effects of SAMe to be evident against APAP-induced hepatotoxicity.

Oxidative stress has been recognized as a major factor in various liver diseases. Hepatocytes play an important role in the metabolism of drugs, and increased drug intake enhances the production of reactive oxygen species in various metabolic processes [18, 19]. Hence, cellular antioxidants are essential for the reduction of oxidative stress and the prevention of the associated pathology [20]. To identify the protective mechanism against the oxidative stress induced by APAP, we examined the expression of three antioxidant genes. Hmox-1 is an essential enzyme involved in heme catabolism, whose antioxidant activity is mediated by all byproducts of heme catabolism. The upregulation of Hmox-1 during oxidative stress is an adaptive mechanism that protects cells against stress conditions. Although there was an increase in Hmox-1 expression after pretreatment with SAMe, it was lower than that of the cells treated with only APAP (Figs. 4A and 4D).

Regulation of cellular reduction/oxidation is an important function of the antioxidant system [21]. The thioredoxin system—composed of NADPH, thioredoxin reductase (ThR), and thioredoxin—protects cells against oxidative stress [22]. Nuclear factor erythroid 2-related factor 2 (Nrf2) is activated in response to oxidative stress following hepatotoxic doses of APAP [23]. Mechanistically, Nrf2 activation leads to the transcriptional activation of antioxidant enzymes, and NQO1, which is responsible for the reduction of cellular quinones, is one of the enzymes that detoxifies NAPQI [24]. In our results, Hmox-1 expression was altered based on Phb1 silencing and APAP concentrations but showed no significant change after pretreatment with SAMe. However, expression of ThR and Nqo1 was influenced not only by changes in Phb1 expression and the APAP dose, but also by pretreatment with SAMe (Fig. 4). These results are consistent with previous reports on the release of antioxidant enzymes from hepatocytes during acute liver injury.

GSH is an important molecule that protects the liver against oxidative stress and damage [25]. As GSH depletion is regarded as a precondition for hepatotoxicity of APAP [26], it is important to understand the protective mechanisms that increase GSH levels [27]. Intracellular GSH concentration was increased after treatment with APAP or SAMe and was affected by Phb1 expression (Fig. 5). Although the GSH concentration increased to protect cells against APAP toxicity, depletion of Phb1 impeded this protective activity. In agreement with this result, the expression of genes related to GSH synthesis was found to increase in the Phb1 HE group after APAP treatment. Since GCL is a major determinant of cellular GSH levels [28], we examined the expression of Gclc and Glcm. In this study, the Phb1 HE group treated with 2 mM of APAP showed an increase in the expression of three genes associated with GSH synthesis. Pretreatment with SAMe further increased these mRNA levels after treatment with 2 mM of APAP in the Phb1 KD groups. In particular, the mRNA levels of Gclc and Gclm in the Phb1 HE group increased by 16-fold and 9-fold, respectively, compared to those of Veh in the control group (Figs. 6E and 6F). This not only suggests that APAP influenced GSH concentration, and the expression of genes related to GSH synthesis depending on the level of Phb1 expression, but that SAMe may also alleviate the effects of APAP.

In summary, our study demonstrates that differences in Phb1 expression levels in AML12 cells and SAMe pretreatment led to protective effects against oxidative stress induced by APAP by increasing the synthesis of enzymes related to APAP metabolism and of antioxidant enzymes that aid GSH synthesis. In addition, Phb1 protects hepatocytes against APAP toxicity even at 50% of wild-type levels. A limitation of this study is that only a single cell line was examined. In the future, the relationship between Phb1 expression and treatment with APAP and SAMe will be explored in diverse cell types such as human cells or immune cells. However, this study is the first to investigate the relationship between hepatotoxicity induced by APAP and Phb1 expression. In conclusion, Phb1 deficiency in normal hepatocytes resulted in altered mRNA levels of APAP metabolism-related genes and antioxidant genes during APAP exposure. SAMe pretreatment alleviated the toxicity caused by APAP by upregulating the expression of antioxidant genes.
